# Outpatient surgical institutions in the rural United States: Trends from 2010 to 2020

**DOI:** 10.1016/j.amjsurg.2025.116188

**Published:** 2025-01-06

**Authors:** Vineeth Amba, Shawn Izadi, Tarun Ramesh, Hao Yu

**Affiliations:** a Department of Health Policy and Management, Harvard T.H. Chan School of Public Health, Boston, MA, USA; b Rutgers Robert Wood Johnson Medical School, Piscataway, NJ, USA; c Department of Surgery, Boston Children’s Hospital, Boston, MA, USA; d Department of Surgery, Oregon Health & Science University, Portland, OR, USA; e Department of Population Medicine, Harvard Medical School and Harvard Pilgrim Health Care Institute, Boston, MA, USA

## Abstract

**Background:**

The volume and proportion of surgeries occurring in outpatient settings has increased. However, the growth and distribution of outpatient surgical institutions, namely ambulatory surgery centers (ASCs) and hospital-based outpatient surgical departments (HOPDs), remains understudied in rural areas.

**Methods:**

We used descriptive statistics and a multivariate logistic regression to assess the growth and distribution of ASCs and HOPDs in rural areas from 2010 to 2020, leveraging the Area Health Resources Files and American Community Survey.

**Results:**

From 2010 to 2020, the number of ASCs in rural counties decreased by 4.9 % (410 vs. 390), and rural HOPDs decreased by 14.3 % (1400 vs. 1200). Completely rural counties were over five times likelier to lack both ASCs and HOPDs (AOR = 5.32; 95 % CI = [4.82–5.89]); p < 0.001).

**Conclusions:**

Outpatient surgical institution access in rural America declined. Policymakers should protect rural HOPDs, promote the creation of ASCs in rural communities, and incentivize surgeons to enter rural practice.

## 1. Introduction

There has been a significant growth in the number of surgeries performed in ambulatory settings in the United States (US), driven by advancements in technology and greater cost-effectiveness when compared to inpatient procedures. More than 60 % of surgeries occurred in the outpatient setting in 2011, compared with 19 % just 30 years prior.^1^ This proliferation of ambulatory surgery also came with an increased ability to handle complex procedures in outpatient facilities.^2^ These factors have led policymakers to pay more attention to outpatient surgical institutions.

Outpatient surgery typically occurs in ambulatory surgery centers (ASCs) and hospital-based outpatient surgical departments (HOPDs), with the utilization of the former rising at a faster pace than the latter. This is likely driven by the ability of ASCs to provide more affordable care and greater convenience for physicians and patients.^1,3–6^ As surgical volume is increasingly concentrated in outpatient settings, equity concerns have been raised about access to these services. Prior studies have documented barriers to ambulatory surgical care in patients who have public insurance, reside in areas with greater socioeconomic disadvantage, and are Black and Hispanic.^7,8^ Similar disparities can be seen in the utilization of ASCs specifically.^9^ Poor access to outpatient surgery can delay elective care, leading to emergency or unplanned procedures, greater complications, and higher healthcare resource burden.^10^ Thus, addressing disparities in the utilization of ambulatory surgery should be a priority.

Residents in rural America may face particular challenges in accessing outpatient surgical care. Reasons for these challenges are multifactorial, and include surgeon shortages and hospital closures. Between 2010 and 2020, the rural-urban gap in surgeon supply grew, a finding that was particularly prominent amongst general surgeons.^11^ Since 2005, there have been 193 hospital closures and conversions in rural areas, which could impact surgical services access.^12^ Given these worsening trends in recent decades, it is important to study how the growth of facilities that provide outpatient surgical care — ASCs and HOPDs — may have been impacted in rural regions.

Despite these challenges, there is a paucity of data assessing trends in the supply of ASCs and HOPDs in these areas. This study aims to fill this gap by analyzing the growth and distribution of ASCs and HOPDs across US counties from 2010 to 2020 and determining characteristics associated with counties that lack access to these institutions.

## 2. Materials and methods

We performed a retrospective cross-sectional analysis of all US counties from 2010 to 2020 utilizing annual, county-level data about ASCs, HOPDs, and general surgeons from the Health Resources and Services Administration’s (HRSA) Area Health Resources Files (AHRF) and county characteristics from the American Community Survey (ACS) 5-year estimates. This study was deemed not human subjects research by the Harvard Pilgrim Health Care Institute Institutional Review Board. This study followed STROBE reporting guidelines for observational studies.

We first analyzed the growth and distribution of ASCs and HOPDs from 2010 to 2020 across all US counties, including rural and completely rural counties. Rural counties were defined in this analysis using the 2013 US Department of Agriculture (USDA) rural-urban continuum codes of 4–9 and completely rural counties were defined using the continuum codes of 8–9. A completely rural county translates to having an urban population of fewer than 5000 with or without adjacency to a metro area. These classifications of rural counties are used within other literature.^11,13^

Then, we conducted a multivariate logistic regression analysis to determine characteristics associated with whether a county had any ASCs and HOPDs in a given year. The regression included state and year fixed effects, controlling for the following county-year level covariates: general surgeons per 100,000 residents, population characteristics (racial/ethnic composition, high school degree, employment, health insurance, median household income), urban-rural status (urban, rural, and completely rural counties), and census region. All tests were 2-sided, used an alpha level of 0.05, and were conducted in Stata version 18.0 (StataCorp; College Station, TX).

## 3. Results

From 2010 to 2020, the total number of ASCs in the US increased by 9.4 % (5300 vs. 5800), compared with a 4.9 % decrease of ASCs within rural counties (410 vs. 390). There was a greater percentage of rural counties without an ASC in 2020 (88.01 %) when compared to 2010 (86.71 %). The vast majority of completely rural counties did not have any ASCs in 2010 (99.53 %) or 2020 (99.37 %). Over the same time period, the number of HOPDs in the US declined by 16.2 % (3700 vs. 3100), with a slightly smaller decline within rural counties of 14.3 % (1400 vs. 1200). Between 2010 and 2020, there was a greater percentage of rural counties without an HOPD (30.75 % vs. 41.49 %). In 2010, 48.36 % of completely rural counties did not have an HOPD. 57.21 % of completely rural counties did not have an HOPD in 2020 (Table 1).

**Fig. 1.**
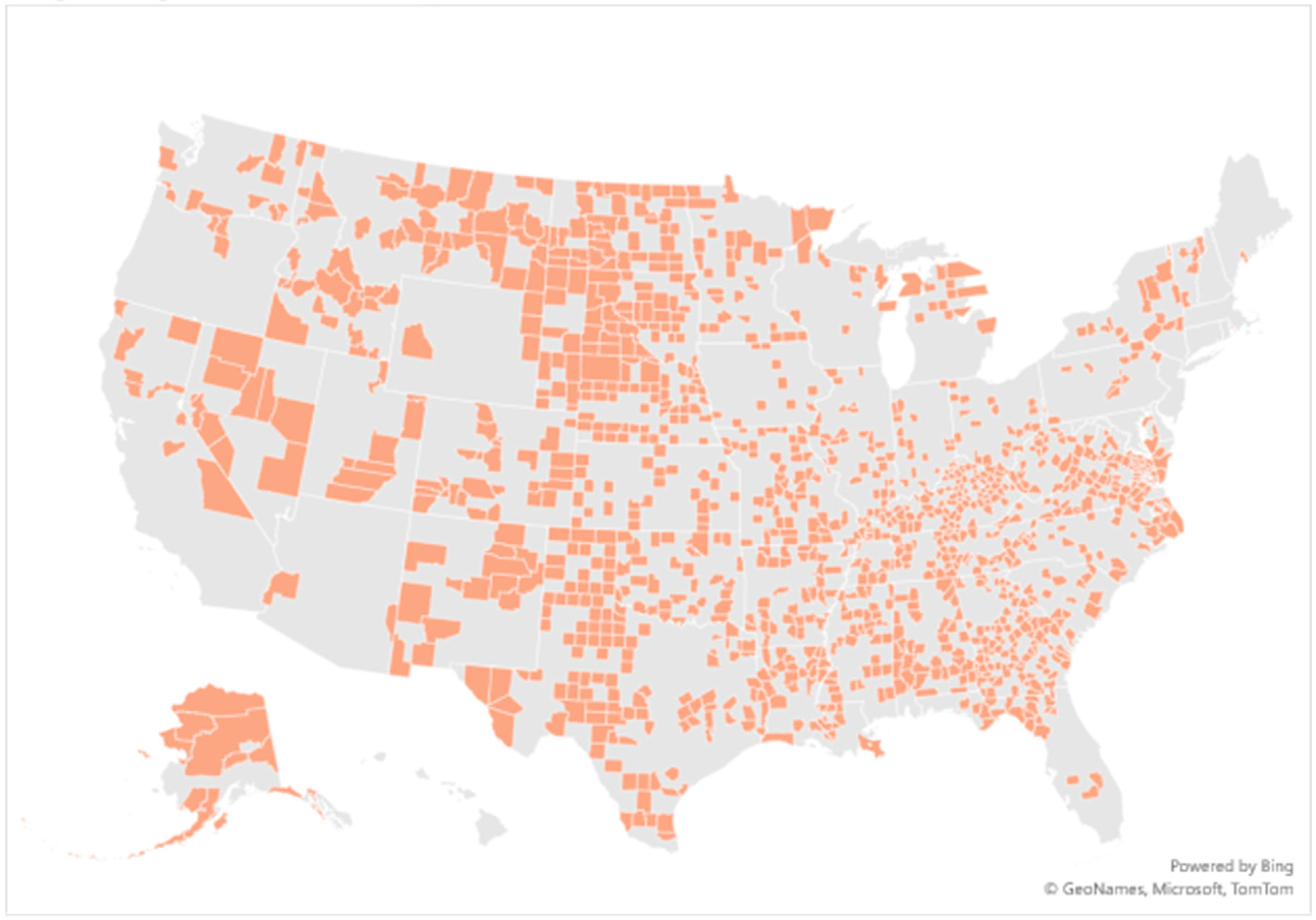
Counties without both ambulatory surgery centers and hospital-based outpatient surgical departments in 2020.^a^ ^a^ This map represents the 1222 counties (38.93 % of US counties) that lack both an ambulatory surgery center and hospital-based outpatient surgical department in 2020. Data about ambulatory surgery centers and hospital-based outpatient surgical departments were extracted from HRSA’s Area Health Resources Files. This map was generated using the Bing Maps visualization integration with Microsoft Excel.

The percent of US counties without both ASCs and HOPDs rose from 31.90 % (1002) in 2010 to 38.93 % (1222) in 2020, the latter visualized in Fig. 1. The number of residents living in such counties increased by 33.4 % between 2010 and 2020 (15,598,113 vs. 20,813,104). In 2010, 37.29 % (or 735) of rural counties were without both ASCs and HOPDs, compared with 46.22 % (910) of rural counties in 2020. The percentage of completely rural counties without both ASCs and HOPDs rose from 72.93 % (466) in 2010 to 79.15 % (505) in 2020 (Table 1).

Communities without both ASCs and HOPDs were more likely to have fewer general surgeons per 100,000 (adjusted odds ratio [AOR] = 0.87; 95 % CI = [0.87–0.88]), a higher percentage of individuals with only a high school degree or equivalent (AOR = 1.08; 95 % CI = [1.07–1.08]), and higher percentages of unemployed (AOR = 1.02; 95 % CI = [1.01–1.04]) and uninsured populations (AOR = 1.02; 95 % CI = [1.01–1.03]). Additionally, for every $10,000 increase in median household income, the odds of not having both an ASC and HOPD in the county decreased by 7 % (AOR = 0.93; 95 % CI = [0.89–0.97]). Such counties were also less likely to be located in the Midwest (AOR = 0.04; 95 % CI = [0.03–0.07]), South (AOR = 0.23; 95 % CI = [0.15–0.37]), and West (AOR = 0.31; 95 % CI = [0.17–0.54]) compared to the Northeast. Completely rural counties were over 5 times more likely to be without both ASCs and HOPDs compared to their non-rural counterparts (AOR = 5.32; 95 % CI = [4.82–5.89]) (Table 2).

## 4. Discussion

Rural communities have long faced prominent obstacles in accessing health services. Our results substantiate these obstacles regarding access to institutions of outpatient surgery. We find that the numbers of ASCs and HOPDs decreased in rural counties from 2010 to 2020. This observed rural trend for ASCs was different from national trends, which showed an increase in the overall number of ASCs. Over this decade, the percentages of rural and completely rural counties without both ASCs and HOPDs increased, revealing deteriorations in access to outpatient surgery in both types of counties. The findings were particularly concerning for the approximately 640 completely rural US counties, which were over five times likelier to not have either type of outpatient surgery facility.

This work extends a previous study by Janeway et al., which found that patients who lived in nonmetropolitan regions were less likely to receive surgical care in ASCs.^9^ Our study complemented this work by including all US counties in our analysis and establishing trends in both ASCs and HOPDs over a more recent time period. The findings of our study suggest that rural-urban disparities in outpatient surgery access enlarged despite initiatives to promote geographic equity on this front.

Our research raises concerns about health consequences stemming from poor access to outpatient surgical centers.^10,14^ The declining numbers of ASCs and HOPDs in rural counties during the past decade, coupled with a greater share of rural and completely rural counties lacking both ASCs and HOPDs compared to the US at large, highlight a common barrier for rural residents — the need to travel farther to receive outpatient surgical treatment. Importantly, research points to longer distances to health services being associated with poorer health outcomes.^15^ Having fewer local ASCs and HOPDs may also cause delays or other access issues for elective care, which can potentially generate negative impacts on patient outcomes. These impacts can be illustrated by conditions commonly treated in outpatient surgical centers. For example, significant delays in cataract surgery can lead to diminished quality of life and vision loss while in the waiting period. Cooper et al. found that delays in hip and knee replacement were associated with joint deterioration.^16,17^

Our findings also raised equity concerns. We found that while the number of ASCs increased in the US from 2010 to 2020, the number of ASCs in rural counties decreased over the same period. This is troubling for multiple reasons. First, costs and procedure time are generally lower for procedures in ASCs, compared with HOPDs.^1,4,6,18^ Second, existing data has pointed towards better post-operative outcomes in ASCs (compared to HOPDs), such as a lower likelihood of inpatient admission and emergency department visit after surgery.^19^ Third, an increasing share of outpatient procedures are occurring in ASCs.^5,20,21^ Considering these factors, it is important to ensure that the growth of ASCs and the ongoing shift of procedural volume towards them do not widen urban-rural disparities in access to surgical care.

We found that counties without both ASCs and HOPDs were more likely to have a greater percentage of the population without insurance coverage. Furthermore, such counties were less likely to have higher median household incomes. These findings are consistent with both qualitative and quantitative evidence that higher poverty and uninsurance rates in rural counties may increase financial strain on hospitals, which could eventually force closures.^22–24^ In addition, areas without outpatient surgical facilities were more likely to have fewer general surgeons per capita. Considering that counties lacking both ASCs and HOPDs were much likelier to be completely rural, this workforce finding aligns with well-established trends of surgeons choosing to not practice in rural areas.^25^ Importantly, this ongoing surgical staffing shortage has the potential to exacerbate closures of rural healthcare centers.^26^

Supporting rural surgeon workforce growth will be crucial in ensuring that ASCs and HOPDs can function in these areas. For example, Congress should pass the “Ensuring Access to General Surgery Act,” which aims to help establish “general surgery shortage areas.”^27^ General surgeons practicing in rural regions could receive incentives like student loan repayments for doing so, and thus, workforce supply may be enhanced. Another avenue to bring surgeons to rural regions is the Health Professional Shortage Area (HPSA) Surgical Incentive Payment Program (HSIP), which offered bonus payments for conducting surgical procedures in provider-shortage regions. Evidence has suggested that this program was associated with a greater count of surgical procedures in HPSAs.^28^ Despite its utility, the program only ran from 2011 to 2015. Federal officials should reinstate this program, considering its success has already been measured.

There are a few policy options that could be considered to maintain access to outpatient surgery institutions for rural communities. Broadly, state and federal policymakers should pursue initiatives to enhance the financial security of rural hospitals with HOPDs. The Affordable Care Act’s (ACA) Medicaid expansion has been associated with improved financial standing and reduced risk of hospital closure, especially in rural areas.^24^ Even so, ten states still have yet to expand their Medicaid programs at the time of writing.^29^ Existing US Department of Health and Human Services grants for financially struggling rural hospitals could also be strengthened. During the Biden-Harris administration, multiple rural hospitals took advantage of these grants to obtain assistance in improving financial security.^30^

Another opportunity to protect rural hospitals is the Rural Emergency Hospital (REH) program. Hospitals that choose to become REHs will receive additional payments by only providing emergency and outpatient healthcare services and forgoing inpatient capabilities.^31^ However, only 32 hospitals have been transformed into REHs since the bill took effect in 2023.^32^ Greater federal assistance in aiding vulnerable, resource-limited rural hospitals in this transition are needed. Further research should evaluate the impact of the REH designation on access to and preservation of outpatient surgical care.

Since the passage of the ACA, ASC reimbursements have been lower than those of HOPDs. Of note, in 2019, the Centers for Medicare and Medicaid Services (CMS) updated ASC payments to rectify this disparity.^21^ Moving forward, it will be important to investigate whether these payment updates influence ASC growth, and whether this growth has helped reduce urban-rural disparities in ASC access. To maintain equitable access to these institutions for rural patients, CMS could consider enhanced payments or other incentives for ASC development in rural regions.

Our study is limited by its retrospective nature, the inability to assess trends at subcounty levels, and missing data or potential mis-classifications of ASCs and HOPDs.

## 5. Conclusions

Our results suggest that rural counties experience enduring barriers to outpatient surgical institution access, and that these trends have only worsened during the past decade. Our findings are especially worrisome since there has been a clear shift of surgical procedures to the outpatient setting, especially ASCs. Federal and state officials should urgently consider policy solutions to promote ASC and HOPD development in rural areas. Further research is needed to evaluate how the new ASC payment reforms and REH program impact the growth of ASCs and HOPDs in rural areas, as well as patient outcomes.

## Figures and Tables

**Table 1:** Changes in ambulatory surgery centers and hospital-based outpatient surgical departments from 2010 to 2020.^a^

Variable	2010	2020	Change, absolute (%)
Total number of ambulatory surgery centers	5300	5800	500 (9.4)
Number of ambulatory surgery centers in rural counties	410	390	−20 (−4.9)
Total number of hospital-based outpatient surgical departments	3700	3100	−600 (−16.2)
Number of hospital-based outpatient surgical departments in rural counties	1400	1200	−200 (−14.3)
Counties without ambulatory surgery centers	2246	2261	15 (0.7)
% of counties without ambulatory surgery centers	71.51	72.03	0.52 (0.7)
Rural counties without ambulatory surgery centers	1709	1733	24 (1.4)
% of rural counties without ambulatory surgery centers	86.71	88.01	1.3 (1.5)
Completely rural counties without ambulatory surgery centers	636	634	−2 (−0.3)
% of completely rural counties without ambulatory surgery centers	99.53	99.37	−0.16 (−0.2)
Counties without hospital-based outpatient surgical departments	1074	1323	249 (23.2)
% of counties without hospital-based outpatient surgical departments	34.19	42.15	7.96 (23.3)
Rural counties without hospital-based outpatient surgical departments	606	817	211 (34.8)
% of rural counties without hospital-based outpatient surgical departments	30.75	41.49	10.74 (35.1)
Completely rural counties without hospital-based outpatient surgical departments	309	365	56 (18.2)
% of completely rural counties without hospital-based outpatient surgical departments	48.36	57.21	8.85 (18.4)
Counties without both ambulatory surgery centers and hospital-based outpatient surgical departments	1002	1222	220 (22.0)
% of counties without both ambulatory surgery centers and hospital-based outpatient surgical departments	31.90	38.93	7.03 (22.0)
Total population living in counties without both ambulatory surgery centers and hospital-based outpatient surgical departments	15,598,113	20,813,104	5,214,991 (33.4)
% of the US population living in counties without both ambulatory surgery centers and hospital-based outpatient surgical departments	5.05	6.31	1.26 (25.0)
Rural counties without both ambulatory surgery centers and hospital-based outpatient surgical departments	735	910	175 (23.8)
% of rural counties without both ambulatory surgery centers and hospital-based outpatient surgical departments	37.29	46.22	8.93 (23.9)
Completely rural counties without both ambulatory surgery centers and hospital-based outpatient surgical departments	466	505	39 (8.4)
% of completely rural counties without both ambulatory surgery centers and hospital-based outpatient surgical departments	72.93	79.15	6.22 (8.5)
Total counties	3141	3139	−2 (−0.1)
Total rural counties	1971	1969	−2 (−0.1)
% of rural counties	62.75	62.73	−0.02 (−0.03)
Total completely rural counties	639	638	−1 (−0.2)
% of completely rural counties	20.34	20.32	−0.02 (−0.10)

a There has been an overall reduction in ambulatory surgery centers and hospital-based outpatient surgical departments in rural areas. Data about ambulatory surgery centers and hospital-based outpatient surgical departments were derived from HRSA’s Area Health Resources Files in 2010 and 2020, while urban-rural status was based on the 2013 USDA rural-urban continuum codes of 1,2,3 representing metropolitan counties, 4,5,6,7,8,9 representing nonmetropolitan or rural counties, and 8 and 9 representing completely rural counties.

**Table 2:** Characteristics of counties without both ambulatory surgery centers and hospital-based outpatient surgical departments.^a^

Variable	Adjusted OR (95 % CI)	*P*-value
Number of General Surgeons (per 100,000)	0.87 (0.87–0.88)	<0.001
% White, non-Hispanic [Reference]	1 [Reference]	NA
% Black, non-Hispanic	1.00 (1.00–1.01)	0.011
% Hispanic	1.00 (0.99–1.00)	0.024
% Other, non-Hispanic	1.00 (0.99–1.00)	0.023
% High School Degree (or Equivalent)	1.08 (1.07–1.08)	<0.001
% Unemployment	1.02 (1.01–1.04)	0.001
Median Household Income (in $10,000)	0.93 (0.89–0.97)	0.001
% Uninsured	1.02 (1.01–1.03)	<0.001
Northeast [Reference]	1 [Reference]	NA
Midwest	0.04 (0.03–0.07)	<0.001
South	0.23 (0.15–0.37)	<0.001
West	0.31 (0.17–0.54)	<0.001
Metropolitan Counties [Reference] (RUCC 1,2,3)	1 [Reference]	NA
Non-metropolitan/Rural (RUCC 4,5,6,7)	0.64 (0.59–0.69)	<0.001
Completely Rural (RUCC 8,9)	5.32 (4.82–5.89)	<0.001

a This multivariate logistic regression shows that counties without both ambulatory surgery centers and hospital-based outpatient surgical departments were less likely to have access to general surgeons. These counties were also more likely to have higher percentages of non-Hispanic Black residents, Hispanic residents, individuals with only a high school degree or equivalent, unemployed individuals and uninsured individuals. Finally, these counties were more likely to be completely rural. The regression included state-year fixed effects. Data about ambulatory surgery centers, hospital-based outpatient surgical departments, and general surgeons per 100,000 were extracted from HRSA’s Area Health Resources Files from 2010 to 2020, while demographic and socioeconomic information were derived from the American Community Survey 5-year estimates at the county level, and data on geography was based on USDA rural-urban continuum codes of 1,2,3 representing metropolitan counties, 4,5,6,7 representing nonmetropolitan or rural counties, and 8 or 9 representing completely rural.
